# Obtention and Characterisation of Gelatine-Based Hydrogels Reinforced with the Amino Acids Cysteine (Cy) and Glutamine (Gt)

**DOI:** 10.3390/gels12050404

**Published:** 2026-05-07

**Authors:** Heidi Fonseca Florido, Ángel Villabona-Ortiz, Rodrigo Ortega-Toro

**Affiliations:** 1 Consejo Nacional de Humanidades, Ciencias y Tecnologías (CONAHCYT), Centro de Investigación en Química Aplicada (CIQA), Saltillo 25294, Mexico; heidi.fonseca@ciqa.edu.mx; 2 Process Design, and Biomass Utilization Research Group (IDAB), Chemical Engineering Department, Universidad de Cartagena, Avenida del Consulado St. 30, Cartagena de Indias 130015, Colombia; avillabonao@unicartagena.edu.co; 3 Food Packaging and Shelf-Life Research Group (FP&SL), Food Engineering Department, Universidad de Cartagena, Avenida del Consulado St. 30, Cartagena de Indias 130015, Colombia

**Keywords:** hydrogels, amino acid reinforcement, cysteine, glutamine

## Abstract

The use of conventional plastics represents a major environmental concern, as approximately 79% ultimately accumulate in landfills or natural ecosystems. Consequently, there is growing interest in the development and application of renewable materials for food packaging. Therefore, the aim of this study was to develop and characterise gelatine-based hydrogels through the incorporation of two amino acids, cysteine and glutamine, thereby contributing to the advancement of safe and environmentally responsible materials. The hydrogels were prepared using the casting method and characterised in terms of their physical and structural properties. The results indicated that the addition of cysteine and glutamine significantly modified the structural, optical, thermal and barrier properties of the gelatine films and hydrogels. Cysteine produced materials with increased opacity, brownish hues and a more hydrophobic surface, changes attributed to the formation of disulphide bonds and the redistribution of non-polar functional groups towards the surface. In contrast, glutamine yielded more transparent and homogeneous films with a more intact internal structure, owing to the development of a more effectively cross-linked and stable polymeric network. Structural (XRD, FTIR) and thermal (TGA, DSC) analyses confirmed that glutamine enhances thermal stability and molecular cohesion, whereas cysteine increases the more ordered structure and rigidity of the matrix. The selection of an appropriate amino acid thus enables the tailoring of functional properties in these biopolymers, representing an effective strategy for their adaptation to biodegradable packaging applications.

## 1. Introduction

The use of conventional plastics represents a major environmental concern, as approximately 79% ultimately accumulate in landfills or natural ecosystems. These materials may require between 100 and 1000 years to degrade and, during this time, release microplastics and toxic additives that adversely affect wildlife, soils and water resources [1]. Consequently, there is growing interest in the development of renewable and biodegradable materials for food packaging applications.

Within this context, hydrogels, three-dimensional networks of hydrophilic polymers, have attracted considerable attention across various sectors, including the agricultural and food industries. These materials are composed of natural or synthetic biodegradable polymers capable of absorbing large quantities of water without losing structural integrity. Hydrogels produced from renewable sources such as gelatine, alginate, starch, or chitosan degrade without generating toxic residues, making them promising alternatives to conventional plastic-based materials used in food packaging systems [2].

Among these biopolymers, gelatine has been widely studied due to its natural origin, biocompatibility, biodegradability, and film-forming ability. Gelatine-based materials exhibit a three-dimensional network capable of retaining substantial amounts of water, which makes them suitable for hydrogel formation and edible films. However, gelatine-based hydrogels often present several limitations, including relatively low mechanical strength, high sensitivity to moisture, and limited barrier properties, which restrict their direct application in food packaging systems. Therefore, different modification strategies have been explored to improve their physicochemical and functional properties [3].

In this regard, amino acids represent promising modifiers for protein-based materials due to their diverse functional groups and ability to interact with polymeric networks. Cysteine and glutamine were selected in this study because of their distinct chemical structures and interaction mechanisms. Cysteine contains a thiol (–SH) group that can participate in intermolecular interactions and potentially promote the formation of cross-linked structures within protein matrices, which may influence the structural stability of the hydrogel network [4]. In contrast, glutamine contains an amide functional group capable of forming hydrogen bonds, which can enhance intermolecular cohesion and contribute to the stabilisation of the gelatine structure. The comparative evaluation of these amino acids therefore provides insight into how different molecular interactions affect the organisation and physicochemical behaviour of gelatine-based hydrogels [5]. Although previous studies have explored the modification of gelatine-based materials using various additives, including amino acids, most reports have focused on individual functional improvements or on biomedical applications. Limited information is available regarding the comparative influence of different amino acids on the structural organisation and physicochemical behaviour of gelatine hydrogels intended for sustainable material applications. In particular, the combined evaluation of structural, optical, thermal and surface properties of gelatine hydrogels modified with cysteine and glutamine has not been widely reported. Therefore, the objective of this study was to develop gelatine-based hydrogels modified with cysteine and glutamine and to systematically evaluate how the incorporation of these amino acids affects the molecular organisation and physicochemical properties of the resulting materials. Understanding these interactions may contribute to the design of biodegradable hydrogel-based materials with improved functionality and potential application in environmentally friendly food packaging systems.

## 2. Results and Discussion

### 2.1. Optical and Colour Properties

The mean values and standard deviations of the colour and gloss parameters of the gelatine hydrogels containing different concentrations of amino acids are presented in Table 1. The incorporation of these compounds influenced the optical properties of the material, which are relevant in determining its visual appearance.

The Gel-C control hydrogel exhibited the highest gloss value (67.43 ± 0.3 GU), indicating a smoother and more reflective surface. The incorporation of amino acids led to a reduction in this parameter, particularly in glutamine-containing hydrogels (Gel-Gt1 and Gel-Gt2), which displayed the lowest gloss values (28.43 and 30.71 GU, respectively). This behaviour may be associated with the formation of additional interactions between gelatine chains and the amino acids, which can modify the organisation of the polymer network and reduce the optical uniformity of the hydrogels [6]. In addition, gloss is strongly influenced by surface morphology. The incorporation of amino acids may promote microstructural rearrangements within the gelatine matrix, leading to an increase in surface irregularities or roughness. Such changes can enhance light scattering at the surface, thereby reducing the measured gloss values. This interpretation is consistent with the SEM observations presented in Section 2.7, where the amino acid-containing hydrogels exhibit a less homogeneous surface morphology compared with the control sample. Regarding colour parameters, all hydrogels exhibited high L* values, indicating a generally light appearance. Glutamine-based hydrogels (Gel-Gt1 and Gel-Gt2) showed the highest lightness values (84.7 and 75.7, respectively). In contrast, cysteine-containing hydrogels, particularly Gel-Cy1, demonstrated a marked increase in the a* and b* coordinates, reflecting enhanced redness and yellowness. This chromatic alteration can be attributed to the formation of coloured compounds resulting from reactions between the thiol groups of cysteine and carbonyl groups present in the system [6]. These changes were accompanied by a decrease in the hue angle (h), from 82.8° in the control sample to 80.3° in Gel-Cy1, indicating a warmer tone associated with cysteine oxidation reactions. Furthermore, an increase in chroma (C), which reflects colour purity or saturation, was observed in cysteine-based formulations, with the highest value recorded for Gel-Cy1 (44.1), confirming the colour intensification induced by this amino acid. Conversely, glutamine-containing films exhibited lower chroma values (13.2 and 40.6), corresponding to a more homogeneous and less saturated appearance. Hue angle values ranging between 80° and 95° indicated a predominance of yellowish tones, with Gel-Cy2 presenting the highest value (95.0°). Total colour difference (ΔE) analysis revealed that Gel-Cy1 underwent the most pronounced variation (12.8), corresponding to a clearly perceptible chromatic change. In contrast, glutamine-based formulations showed significantly lower ΔE values (between 5.3 and 8.6), suggesting a considerably milder effect on the overall visual appearance.

Overall, cysteine exerted a more pronounced influence on colour and gloss, producing warmer and more vivid tones. By contrast, glutamine promoted the formation of lighter films with lower gloss and a more uniform appearance. These variations arise from the distinct chemical structures of the amino acids and their different modes of interaction within the gelatine protein matrix during film formation.

### 2.2. Transmittance and Opacity

**Figure 1:**
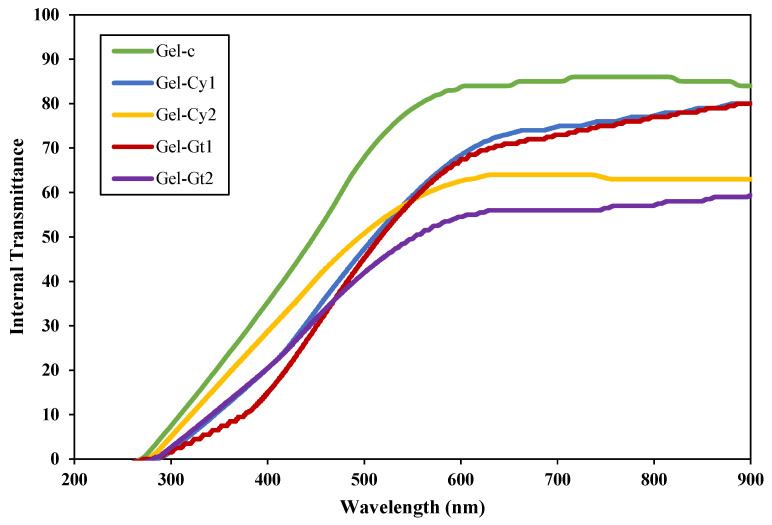
UV–Vis direct transmittance spectra of the different treatments.

Figure 1 presents the UV–Vis transmittance spectra (200–900 nm) of gelatin-based hydrogels reinforced with cysteine (Cy) or glutamine (Gt). Transmittance is directly related to the amount of light passing through the hydrogels and therefore reflects the transparency of the obtained materials [7]. In this regard, all hydrogels exhibited low transmittance in the ultraviolet region (<300 nm), indicating high absorption within this range, which is typical of protein-based systems containing chromophoric groups associated with peptide bonds and other organic components. The incorporation of amino acids led to a noticeable reduction in transmittance across the visible region, suggesting modifications in the structural organisation and homogeneity of the gelatin network. The Gel-Cy2 and Gel-Gt2 samples were the most affected, showing transmittance values of only 55–60% above 600 nm. This decrease in transparency can be attributed to an increase in light scattering within the hydrogel matrix. The incorporation of amino acids may induce microstructural heterogeneities, such as localised domains, changes in polymer chain packing, or small-scale phase irregularities, which disrupt the uniform propagation of light through the material.

Furthermore, higher amino acid concentrations (Cy2 and Gt2) resulted in a more pronounced decrease in transmittance compared with their lower-concentration counterparts (Cy1 and Gt1), indicating that the additive content plays an important role in determining optical clarity. The greater density of intermolecular interactions between gelatin chains and amino acid molecules may promote structural rearrangements within the polymer network, leading to a less homogeneous microstructure. These interpretations are consistent with the SEM observations discussed in Section 2.7, where the amino acid-containing hydrogels exhibit a more heterogeneous surface morphology compared with the control sample. Such microstructural features can enhance light scattering and therefore contribute to the observed reduction in transparency. Overall, these results demonstrate that the incorporation of cysteine and glutamine modifies the optical properties of gelatin hydrogels by affecting the structural homogeneity and microstructural organisation of the polymer network, which in turn influences light transmission through the gel matrix [8].

**Figure 2:**
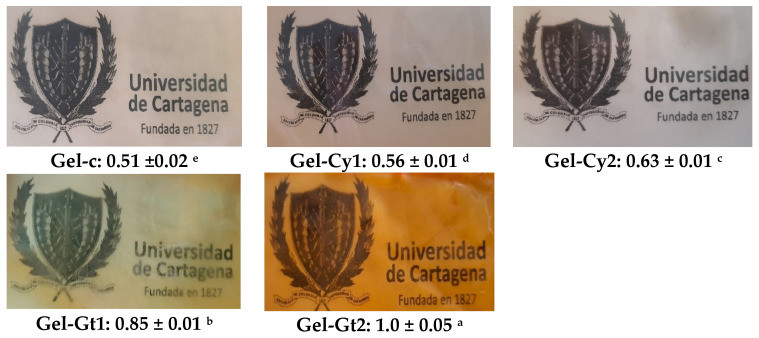
Appearance of the films as shown in photographs, and mean values ± standard deviation of the opacity of the studied films. Different superscript letters indicate significant differences (*p* < 0.05) between the formulations.

Figure 2 illustrates the visual appearance and opacity values of gelatine-based hydrogels reinforced with cysteine (Cy) or glutamine (Gt). Opacity is defined as the amount of light blocked or absorbed by the film and is inversely related to transmittance [9]. The control film (Gel-C) was the most transparent and glossy, exhibiting the lowest opacity value (0.51). This behaviour is consistent with the photographic image shown in Figure 2, where the control film appears clearer and allows greater visual transmission of light through the material. In contrast, the incorporation of cysteine progressively increased opacity (Gel-Cy1 = 0.56; Gel-Cy2 = 0.63), reduced gloss and introduced yellowish tones, suggesting chemical interactions between thiol groups and the gelatine matrix [10]. These changes are also visually evident in the corresponding images, where the films appear slightly less transparent than the control. However, glutamine produced the most pronounced effect, with opacity values ranging from 0.85 to 1.0, resulting in significantly denser and less transparent films. This is clearly reflected in the photographic images, where glutamine-containing films appear opaquer and allow less light to pass through the material. This behaviour is consistent with the reduction in gloss and colour saturation reported in Table 1.

Overall, the results obtained for transmittance, opacity and colour are physically consistent, as all these parameters depend on the interaction of light with the internal structure of the hydrogel films. Variations in the organisation of the gelatine network, induced by the incorporation of cysteine and glutamine, can modify the way light propagates through the material. Structural heterogeneities may increase light scattering within the matrix, which simultaneously reduces transmittance, increases opacity and influences the perceived colour of the films. Therefore, the changes observed in these optical parameters can be interpreted as complementary evidence of the structural modifications occurring within the gelatine-based hydrogel network.

### 2.3. Physical Properties and Water Absorption

Table 2 presents the mean values and standard deviations of the physical and water absorption properties of gelatine-based hydrogels reinforced with cysteine (Cy) or glutamine (Gt). The parameters analysed included thickness (μm), water vapour permeability (WVPt), swelling degree (Hwg), moisture content (Xwg) and water absorption capacity (Wcag) of the evaluated films. The thickness of the obtained hydrogels ranged from 215.7 μm to 351.8 μm, with statistically significant differences (*p* < 0.05) observed depending on the incorporated amino acid. Hydrogels containing amino acids exhibited greater thickness than the control sample (Gel-C), particularly Gel-Cy2. This behaviour may be attributed to additional intermolecular interactions promoted by the functional groups of the amino acids, which favour the formation of a more expanded polymeric network [11]. Regarding water vapour permeability (WVPt), no significant differences were observed among the hydrogels, indicating that the presence of cysteine or glutamine did not substantially alter the vapour barrier properties. Nevertheless, the slight decrease observed in Gel-Gt2 may be associated with a denser structure or more effective cross-linking, which could hinder water vapour diffusion through the matrix [12]. In terms of swelling capacity (Hwg), cysteine-containing hydrogels (Gel-Cy1 and Gel-Cy2) exhibited higher water uptake. This behaviour may be associated with the presence of sulfhydryl (–SH) groups, which can increase the hydrophilic character of the polymer network and promote interactions with water molecules through polar interactions. As a result, the incorporation of cysteine may enhance the affinity of the hydrogel matrix for water, favouring greater swelling. Conversely, glutamine-based hydrogels (Gel-Gt1 and Gel-Gt2) showed lower swelling values, which may indicate the formation of a more compact and organised polymer network. The amide functional groups of glutamines can promote hydrogen bonding between polymer chains, increasing intermolecular cohesion and reducing the free volume available for water diffusion. With respect to water absorption capacity (Wcag), formulations containing cysteine displayed lower absorption compared with those incorporating glutamine. This behaviour may be related to differences in the balance between hydrophilic interactions and network compactness. Although cysteine may enhance water affinity at the molecular level, the structural rearrangements induced by its incorporation may limit the retention of water within the matrix. In contrast, glutamine-containing hydrogels may retain water more effectively due to the presence of amide groups capable of forming hydrogen bonds with water molecules. Finally, moisture content (Xwg) remained relatively stable across formulations, except for Gel-Gt2, which recorded the highest value (0.118 g water/g dry film). This result may be related to its greater thickness and the formation of a denser polymer network capable of retaining water molecules within the hydrogel structure.

Overall, the combined results obtained from TGA, DSC, XRD, FTIR and SEM analyses provide a comprehensive understanding of the structural modifications induced by amino acid incorporation into the gelatine matrix. Thermal analyses revealed that formulations containing cysteine and glutamine exhibited improved thermal resistance, particularly Gel-Cy2 and Gel-Gt2, indicating enhanced intermolecular cohesion within the polymer network. These findings are consistent with the XRD results, which showed slight shifts in diffraction peaks, suggesting modifications in molecular packing and structural organisation of the gelatine chains. Similarly, FTIR spectra confirmed the presence of additional intermolecular interactions, including hydrogen bonding promoted by glutamine and possible disulphide bridge formation associated with cysteine. These structural rearrangements were further supported by SEM observations, where amino acid-containing hydrogels exhibited noticeable differences in morphology, pore distribution and network compactness compared with the control sample. In particular, the denser and more homogeneous structure observed in Gel-Gt2 correlates well with its higher thermal stability and reduced porosity. Therefore, the incorporation of cysteine and glutamine not only modifies the chemical environment of the gelatine matrix but also induces structural reorganisation at both molecular and microstructural levels, which ultimately influences the thermal and physicochemical behaviour of the developed hydrogels.

### 2.4. Contact Angle

Table 3 presents the water contact angle (CAw) and oil contact angle (CAo) values of gelatine-based hydrogels reinforced with cysteine (Cy) or glutamine (Gt). This analysis provides information on the surface wettability and the hydrophilic or hydrophobic behaviour of the studied hydrogels. The contact angle values showed significant differences (*p* < 0.05) depending on the incorporated amino acid. Gel-Cy2 (66.7 ± 1.1°) exhibited the highest CAw value, indicating a lower affinity for water compared with the other formulations. This behaviour may be related to modifications in the surface chemistry of the gelatine matrix induced by cysteine incorporation. Gelatine naturally contains several polar functional groups, such as hydroxyl (–OH), amino (–NH_2_) and carboxyl (–COOH), which favour hydrogen bonding with water molecules and promote surface hydrophilicity. When cysteine is incorporated into the matrix, the presence of sulphydryl (–SH) groups may alter the distribution and orientation of functional groups during film formation. These thiol groups exhibit lower polarity compared with hydroxyl or carboxyl groups and may preferentially orient towards the surface of the film. As a result, the number of highly polar sites available for hydrogen bonding with water decreases, leading to an increase in the water contact angle and a more hydrophobic surface character [13]. Conversely, the control hydrogel (Gel-C) displayed the lowest CAw value (52.0°), indicating a more hydrophilic surface with greater affinity for water. Regarding CAo values, a higher oil contact angle (as observed for Gel-Gt2 and Gel-Cy1) corresponds to a less oleophilic and more polar surface, favouring interactions with polar phases. In contrast, lower CAo values (Gel-Cy2 and Gel-C) indicate a more oleophilic and relatively hydrophobic surface. This behaviour may be associated with the preferential orientation of less polar functional groups, such as cysteine thiol groups, towards the interface, which can increase compatibility with non-polar compounds [14].

Overall, the contact angle results are consistent with the water-related properties and microstructural observations of the hydrogels. Samples showing higher water contact angles tended to exhibit lower swelling capacity and reduced water uptake, suggesting a decrease in surface hydrophilicity. Conversely, hydrogels with lower contact angles displayed greater affinity for water, which is reflected in their greater swelling behaviour. These trends are also supported by the SEM observations, where differences in network compactness and pore distribution were identified among the formulations. Therefore, the contact angle analysis complements the swelling and microstructural results, indicating that the incorporation of amino acids modifies both the surface wettability and the internal organisation of the gelatine hydrogel matrix.

### 2.5. Cumulative Weight Loss

**Figure 3:**
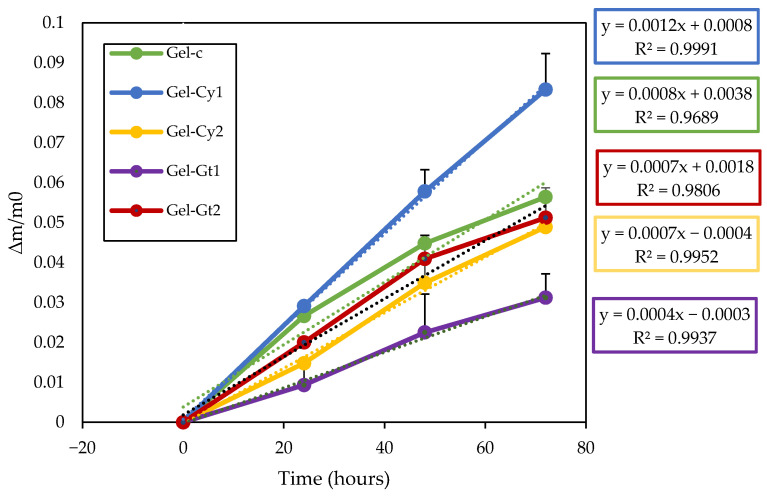
Cumulative weight loss of the studied films stored at ambient temperature (25 °C). The dotted lines indicate the trend.

Figure 3 reveals differences in film stability during storage at ambient temperature, assessed through cumulative weight loss. Although all formulations exhibited progressive moisture loss, the dehydration rate varied according to composition. The Gel-Cy1 film, containing a low concentration of cysteine, showed the fastest dehydration, suggesting a less cohesive polymeric network with lower structural stability. This behaviour may be associated with partial disruption of hydrogen bonding interactions within the gelatine matrix, which can reduce the capacity of the hydrogel to retain water molecules during storage. In contrast, Gel-Gt2, with a higher glutamine content, demonstrated the greatest stability and the lowest cumulative weight loss. The amide functional groups present in glutamine can promote additional hydrogen bonding with gelatine chains, contributing to the formation of a more compact and cohesive network capable of retaining moisture more effectively. The remaining formulations (Gel-C, Gel-Cy2 and Gel-Gt1) displayed intermediate stability, confirming that both the nature and concentration of the incorporated amino acid influence water retention and the structural integrity of the films during storage [15]. Glutamine does not introduce thiol (-SH) groups or promote disulphide bond formation, which may otherwise generate internal stresses or network heterogeneity. Specifically, glutamine promotes the formation of a more cohesive and compact network through hydrogen bonding interactions between its amide groups and gelatine chains, which enhances water retention and reduces weight loss during storage. Its incorporation is therefore more uniform and homogeneous, contributing to a more compact, stable and less brittle structure [16]. In contrast, cysteine, particularly at lower concentrations, may partially disrupt hydrogen bonding due to the presence of thiol groups, resulting in a less compact structure with higher water mobility and faster dehydration. At higher concentrations, cysteine can contribute to network stabilisation through potential disulphide bond formation, improving structural integrity. Furthermore, the coefficients of determination (R^2^) obtained from both plots were notably high, indicating a strong linear fit of the experimental data.

### 2.6. Thermal and Crystalline Properties: Study by Thermogravimetric Analysis (TGA), Differential Scanning Calorimetry (DSC), and X-Ray Diffraction (XRD)

**Figure 4:**
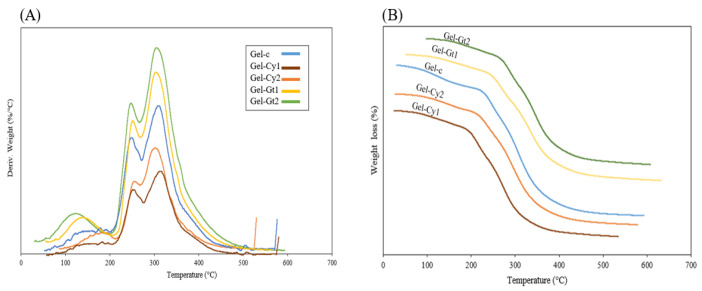
Non-derivative (TGA (**A**)) and derivative (DTG (**B**)) thermogravimetric analysis of gelatine-based hydrogels containing different concentrations of amino acids (Cy and Gt).

Thermal stability analysis of gelatine films incorporating cysteine (Cy) and glutamine (Gt), evaluated by thermogravimetric analysis (TGA) and derivative thermogravimetry (DTG) (Figure 4 and Table 4), revealed three principal stages of decomposition. The first stage (Td_1_), occurring between approximately 100 °C and 160 °C, corresponds to the evaporation of free and bound water physically retained within the hydrogel matrix. This weight loss is commonly associated with the release of adsorbed moisture and weakly bound water molecules interacting with hydrophilic groups of gelatine, such as hydroxyl, amino and carboxyl groups [17]. The second stage (Td_2_), observed between 200 °C and 300 °C, is attributed to the initial thermal degradation of the organic matrix. In this region, the cleavage of peptide bonds and the breakdown of side chains of amino acid residues occur, leading to the progressive degradation of the gelatine polymer structure. Finally, the third stage (Td_3_), located between 300 °C and 400 °C, corresponds to the maximum degradation of the polymeric network, involving extensive decomposition of the protein backbone and the formation of low-molecular-weight volatile compounds. Similar three-stage degradation patterns have been reported for other gelatine-based and protein-derived hydrogel systems [18]. Among the formulations, Gel-Gt2 displayed the highest thermal stability, showing the lowest mass loss compared with the other hydrogels. This behaviour suggests that glutamine promotes the formation of a more cohesive and compact network through hydrogen bonding and amide interactions, which can increase intermolecular cohesion and delay the thermal decomposition of the material [19].

The derivative DTG curve represents the rate of weight loss of the hydrogels as a function of temperature and allows the identification of maximum degradation peaks. As shown in Table 4, the control hydrogel (Gel-C) exhibited a Td_3_ value of 303.60 °C, whereas the incorporation of amino acids resulted in an increase in the maximum degradation temperature. In particular, Gel-Cy2 showed the highest Td_3_ value (314.76 °C), representing an increase of approximately 11 °C compared with the control sample. Similarly, Gel-Gt2 and Gel-Cy1 exhibited Td_3_ values of around 309 °C, indicating an improvement in thermal stability of about 5–6 °C relative to Gel-C. These results demonstrate that the incorporation of cysteine and glutamine contributes to enhancing the thermal resistance of the gelatine matrix. Furthermore, the lower peak intensities observed for these formulations suggest a reduced decomposition rate, implying greater internal cohesion within the polymer network. This behaviour may be associated with the formation of additional intermolecular interactions, such as potential disulphide linkages promoted by cysteine and hydrogen bonding interactions involving glutamine amide groups.

**Figure 5:**
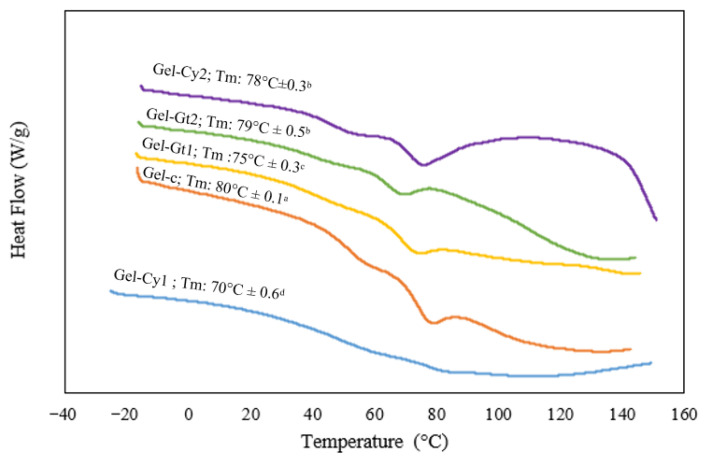
DSC thermograms of gelatine-based hydrogels containing different concentrations of amino acids (Cy and Gt).

The DSC thermograms of gelatine-based hydrogels reinforced with cysteine or glutamine are presented in Figure 5. A single endothermic peak was observed, which is associated with the transition from a more ordered phase to an amorphous state, resulting from the disruption of collagen triple helices [20]. The control hydrogel (Gel-C) exhibited the highest melting temperature (Tm), reaching 80 °C. This elevated value indicates superior thermal stability, attributed to the natural cross-linking present in pure gelatine. In contrast, Gel-Cy1 showed the lowest Tm (70 °C). This reduction suggests that a low concentration of cysteine may disrupt hydrogen bond formation, leading to a polymeric network with lower cohesion and, consequently, reduced thermal stability. The incorporation of cysteine (Gel-Cy2) or glutamine (Gel-Gt1 and Gel-Gt2) increased the melting temperature to a range of 75–79 °C. This enhancement indicates that both amino acids promote intermolecular interactions within the gelatine matrix. In the case of glutamine, its amide groups can form additional cross-linking interactions that reinforce the protein structure, thereby improving thermal stability. These findings are consistent with previous studies reporting that increased cross-linking density in gelatine correlates with higher Tm values and enhanced heat resistance [21]. Overall, DSC analysis demonstrated that the thermal stability of gelatine hydrogels increases with glutamine incorporation and, to a lesser extent, with cysteine addition. This effect is attributed to improved cross-linking and structural cohesion induced by these amino acids. Furthermore, the absence of glass transition and crystallisation events in the thermograms confirms the amorphous nature of the materials, characterised by a cross-linked and plasticised network structure.

**Figure 6:**
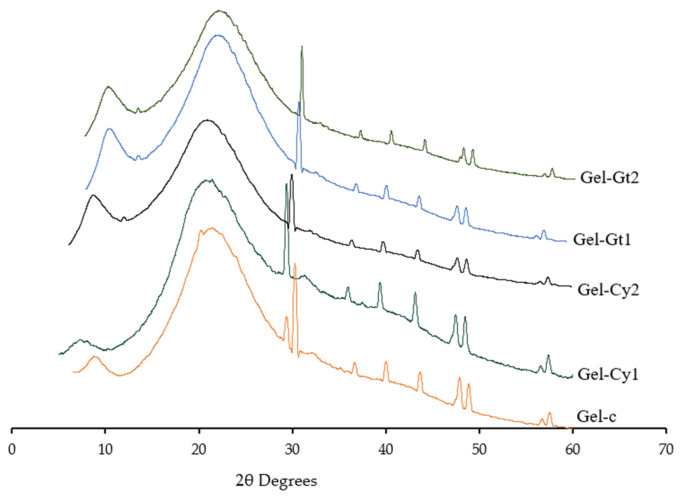
X-ray diffraction patterns of the studied hydrogels.

The X-ray diffraction (XRD) patterns of gelatin-based hydrogels formulated with different concentrations of cysteine (Cy) and glutamine (Gt) are presented in Figure 6 and Table 5. This analysis provides a qualitative assessment of structural organisation within the hydrogels. In general, sharp and intense reflections are associated with highly ordered crystalline domains, whereas broad and diffuse diffraction features are characteristic of predominantly amorphous materials. In the present study, all formulations exhibited mainly broad diffraction profiles, indicating that the gelatin-based matrices were predominantly amorphous, with only limited short-range ordering.

All samples showed two main diffraction features: a low-intensity maximum at approximately 2θ ≈ 11–12° and a broader halo in the region of 2θ ≈ 20–21°. These features are commonly reported in gelatin-based systems and have been associated with residual local helical organisation and the amorphous arrangement of protein chains, respectively. Because these signals were broad and partially overlapping, their positions were determined by peak fitting rather than by direct visual inspection of the diffractograms [22,23].

The incorporation of cysteine and glutamine produced slight but consistent shifts in the position of the broad maximum centred around 20–21°. The control sample (Gel-C) exhibited this feature at approximately 21.0°, whereas Gel-Cy1 and Gel-Cy2 showed a displacement towards lower angles (around 20.0°), suggesting a small increase in intermolecular spacing or a reduction in chain packing density. In contrast, Gel-Gt1 and Gel-Gt2 showed this maximum at slightly higher angles (20.5° and 20.8°, respectively), which may indicate a somewhat more compact local arrangement of the polymeric network [24].

Additional weak reflections were also observed in the higher-angle region, particularly around 37–58° (2θ). These signals varied among formulations, indicating differences in local short-range organisation induced by amino acid incorporation. For example, the shift observed in Gel-Gt2 towards higher 2θ values in the region around 43–45° may be consistent with a more compact local arrangement compared with the control and cysteine-containing samples. However, given the broad nature of the diffraction profiles and the predominantly amorphous character of the materials, these features were not assigned to specific crystalline phases or to individual (hkl) planes.

The XRD results should be interpreted as evidence of subtle structural rearrangements within the gelatin network rather than as proof of a well-defined crystalline structure. Overall, the diffraction results suggest that glutamine favoured a more cohesive and compact molecular arrangement, whereas cysteine induced different rearrangements depending on concentration. These observations are consistent with the FTIR, DSC, TGA and SEM analyses, which also indicate that amino acid incorporation modified intermolecular interactions and matrix organisation.

**Figure 7:**
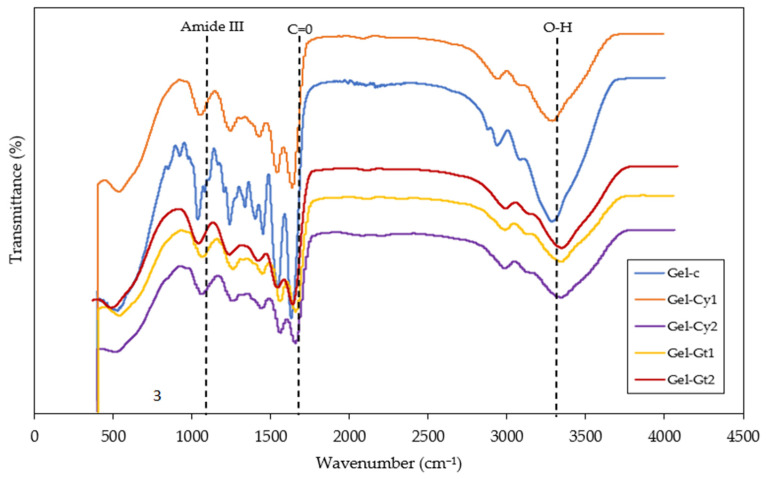
Fourier transform infrared (FTIR) spectra of the studied hydrogels.

Figure 7 and Table 6 present the FTIR analysis of hydrogels reinforced with cysteine (Cy) and glutamine (Gt). FTIR spectroscopy enables the identification of the functional groups present in the studied hydrogels. Broad absorption bands were observed in the range of 3100–3500 cm^−1^, which are mainly associated with O–H stretching vibrations related to hydrogen bonding interactions. However, in gelatine-based systems this region may also include contributions from N–H stretching vibrations (Amide A band), characteristic of peptide structures. The overlap of O–H and N–H stretching vibrations results in the broad profile typically observed in protein-based materials. The intensity of this band was more pronounced in Gel-Gt1 and Gel-Gt2, suggesting enhanced intermolecular interactions through hydrogen bonding within the polymer network. These findings are consistent with previous reports [18], where a broad band around 3400 cm^−1^ was attributed to overlapping O–H and N–H stretching vibrations in gelatine-based materials. Additionally, bands were detected in the range of 1600–1800 cm^−1^, corresponding to the Amide I region (C=O stretching). A slight shift towards lower wavenumbers was observed in hydrogels containing amino acids, indicating the formation of new intermolecular interactions and an increased degree of structural cohesion within the polymer network. Slight variations in the intensity and position of these bands among the different formulations suggest that the incorporation of cysteine and glutamine influences the intermolecular interactions within the gelatine matrix. These changes may be related to hydrogen bonding and possible interactions involving the thiol groups of cysteine and the amide functional groups of glutamines. Although the formation of disulphide bonds cannot be directly confirmed from FTIR analysis alone, the observed spectral variations indicate modifications in the molecular environment and structural organisation of the gelatine network.

The Amide II band (between 1560 and 1520 cm^−1^), associated with N–H bending and C–N stretching vibrations, exhibited lower intensity in cysteine-containing gels. This behaviour may indicate that the presence of cysteine influences the local molecular environment of the gelatine network, possibly affecting intermolecular interactions involving peptide groups [25]. In particular, the sulphydryl (–SH) groups of cysteine may participate in additional interactions within the matrix, which could contribute to modifications in the structural arrangement of the polymer chains. Overall, the results indicate that glutamine acts as a structural stabiliser by promoting hydrogen bond formation, whereas cysteine modifies the molecular network through structural rearrangements and the potential formation of disulphide bridges. This behaviour is consistent with the previous DSC and XRD analyses, in which glutamine-containing samples demonstrated a more ordered structure and enhanced thermal stability.

### 2.7. Microstructure

**Figure 8:**
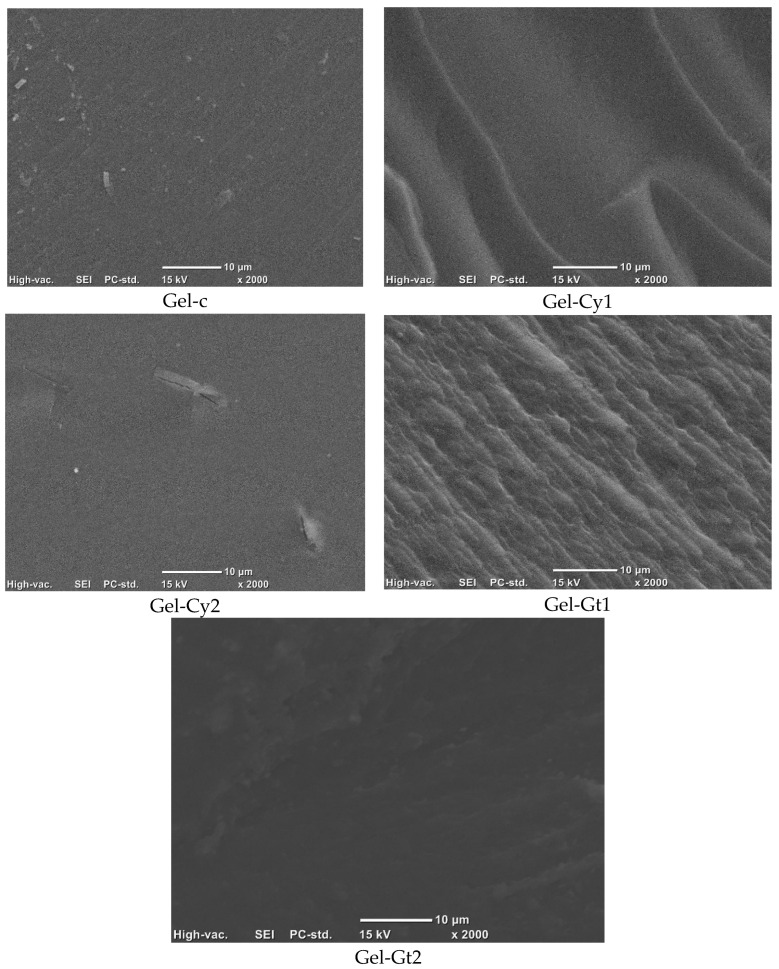
SEM micrographs of the cross-sections of the studied films observed at 2000× magnification.

Cross-sectional micrographs of all formulated samples at magnifications of 2000×, 1500× and 1000× are presented in Figure 8. Cross-sectional micrographs of all formulated samples at 2000× magnification reveal clear differences in the morphology of the modified gelatine hydrogels. The control hydrogel (Gel-C) exhibits a uniform and compact structure without visible pores or cracks, characteristic of unmodified gelatine systems dominated by hydrogen bonding interactions. In contrast, Gel-Cy1 displays a less homogeneous morphology, with the presence of pores, cavities, and fine cracks, indicating disruption of the polymeric network and weakened intermolecular interactions due to the incorporation of cysteine at low concentration. This effect is mitigated in Gel-Cy2, where the structure appears denser, more cohesive, and less porous, suggesting that higher cysteine content promotes structural reorganisation through covalent cross-linking, most likely via disulphide bond formation. Hydrogels containing glutamine (Gel-Gt1 and Gel-Gt2) exhibit more homogeneous, continuous, and well-organised networks. In particular, Gel-Gt2 presents the densest and most compact structure, with reduced porosity and thicker network walls, indicating enhanced intermolecular interactions and effective cross-linking within the gelatine matrix. These morphological observations are consistent with XRD, FTIR, and DSC analyses, which confirm improved structural cohesion and thermal stability in glutamine-modified samples [16].

These microstructural differences are consistent with the functional properties observed in the hydrogels. Samples exhibiting more porous and heterogeneous structures, such as Gel-Cy1, facilitate water penetration into the polymer network, which may contribute to the higher swelling behaviour observed in these formulations. In contrast, the denser and more compact structures identified in Gel-Cy2 and particularly Gel-Gt2 can limit water diffusion and enhance structural cohesion, which is consistent with their lower swelling capacity and improved thermal stability reported in the TGA and DSC analyses. Moreover, variations in surface homogeneity and pore distribution may also influence light scattering within the films, contributing to the differences in opacity observed among the formulations.

## 3. Conclusions

The incorporation of cysteine and glutamine significantly influenced the structural, optical, thermal, and physicochemical properties of gelatine-based hydrogels, with effects depending on both amino acid type and concentration. Optical results showed reduced transmittance and increased opacity after amino acid incorporation, consistent with changes in colour and gloss. These variations are associated with increased light scattering caused by structural heterogeneities within the polymer network. In this regard, cysteine induced more pronounced colour changes, while glutamine produced lighter and more uniform films. These findings are consistent with microstructural and physicochemical results. SEM analysis revealed that cysteine, particularly at low concentration, generated more heterogeneous structures, whereas glutamine promoted more compact and homogeneous networks. This structural organisation explains the observed differences in swelling, water retention, and optical behaviour. Thermal analyses (TGA and DSC) confirmed improved stability in amino acid-containing hydrogels, especially at higher concentrations, which is in agreement with the increased intermolecular interactions identified by FTIR and XRD. Overall, the results are mutually consistent: more compact structures (e.g., glutamine-based hydrogels) exhibited lower swelling, higher opacity, and greater thermal stability, while more heterogeneous systems (e.g., low cysteine content) showed the opposite behaviour. These findings demonstrate that amino acids are effective modifiers for tuning the structure and properties of gelatine-based hydrogels.

## 4. Materials and Methods

### 4.1. Materials

Gelatine type B (food grade) was purchased from local retail stores in the city of Cartagena, Colombia, supplied by Districondorito, and used without further purification. Glutamine was supplied by Alfa Aesar (Ward Hill, MA, USA). Cysteine was obtained from PANREAC (Barcelona, Spain). Finally, glycerol and glutaraldehyde were supplied by Panreac (Bogotá, Colombia).

### 4.2. Preparation of Hydrogels

The Hydrogels were obtained following the procedure reported by [26], with slight modifications. The different formulations evaluated in this study are presented in Table 7. Briefly, gelatine (Type B, food grade) was dissolved in distilled water to obtain a 10% (*w*/*v*) solution under continuous magnetic stirring at 50 ± 2 °C until complete dissolution. Subsequently, the corresponding amino acids (cysteine or glutamine) were added at the concentrations specified in Table 7, and the mixture was stirred for an additional 15 min to ensure complete homogenisation. Glycerol was then incorporated as a plasticising agent at 15% (*w*/*w*) relative to the gelatine content. The mixture was maintained under stirring for 15 min to ensure uniform distribution of the plasticiser. Afterward, glutaraldehyde was added dropwise as a crosslinking agent under continuous stirring. The reaction mixture was maintained under magnetic stirring for 4 h at 50 °C to promote crosslinking and network formation. Finally, the resulting hydrogel solution was poured into casting moulds and dried in a convection oven at 50 °C until constant weight was reached. The dried films were then conditioned at room temperature (25 ± 2 °C) for 24 h prior to further characterisation.

### 4.3. Characterisation of the Films

#### 4.3.1. Colour

The surface colour of the films was evaluated using a portable colorimeter (Colorimeter CHN Spec CS-10, CHNSpec Technology Co., Ltd., Shenzhen, China), which provided the CIE Lab* coordinates, hue angle (h), and chroma or saturation (c). In the CIE Lab* system, lightness (L*) is represented on the vertical axis, while the horizontal axes (a* and b*) indicate the direction toward colours such as red, green, blue, and yellow [27]. Following this, the colour of the films was compared against a reference standard to quantify the chromatic variation. To achieve this, the differences for each colour coordinate (ΔL, Δa, and Δb) were calculated. Subsequently, the total colour difference (ΔE) was derived by applying the appropriate equation.


(1) $$∆E=\sqrt{{∆L}^{*2}+{∆a}^{*2}+{∆b}^{*2}}$$

#### 4.3.2. Gloss

Gloss was determined at an incidence angle of 60°, according to the method described by [28]. A gloss meter featuring a flat surface configuration (3NH YG268 multi-angle gloss meter, Minolta, Langenhagen, Germany) was employed for the analysis. A total of five films were assessed, with seven replicate measurements recorded for each individual sample. The results were subsequently expressed as gloss units (GU).

#### 4.3.3. Opacity

The opacity of each film was determined using a spectrophotometer (BIOBASE BK-UV1900PC UPR19G0005, Biobase Biodustry (Shandong) Co., Ltd., Jinan, China) [29]. Opacity was determined according to Equation (2):


(2) $$Opacity=\frac{Absorbance\;(600\;nm)}{Thickness\;(mm)}$$

#### 4.3.4. Transmittance

For the analysis of internal transmittance, a spectrophotometer (BIOBASE BK-UV1900PC UPR19G0005) fitted with quartz cuvettes was employed. Film specimens measuring 1 × 3 cm were positioned within the quartz cuvettes, and absorbance measurements were subsequently recorded across the UV-visible range [29].

#### 4.3.5. Thickness

Thickness measurements were performed using a digital micrometer (TOP EU TL268, HongKong, Proster Trading Limited, Hong Kong). For each film, seven random readings were recorded, and the mean value, along with the standard deviation, was subsequently calculated.

#### 4.3.6. Moisture Content (Xw) and Swelling Degree (Hw) of the Hydrogels

The methodology proposed by [30] was implemented. Composite film samples (2 cm × 2 cm) were initially weighed to record their starting mass (W_1_) and subsequently dried in an oven maintained at 60 °C until a constant mass (W_2_) was attained. The samples were then immersed in 25 mL of water at room temperature. Following a 24 h incubation period, the water was decanted, and the samples were gently blotted dry with filter paper prior to reweighing (W_3_).


(3) $$Moisture\;content=\frac{W1-W2}{W1}$$

(4) $$Swelling=\frac{W3-W2}{W2}$$

#### 4.3.7. Water Absorption Capacity (WCA)

The water absorption capacity was determined following the methodology described by [31]. Sample preparation involved cutting the films into 2 × 2 cm pieces, which were subsequently placed in a desiccator containing calcium chloride to maintain 0% relative humidity at ambient temperature. The mass of the samples was recorded at 24 h intervals until a constant value was attained; this was designated as the dry film mass (W_s_). Upon achieving stability, the specimens were transferred to a desiccator containing a saturated potassium sulphate solution. The samples were again weighed every 24 h until the film mass stabilised, with this final value recorded as the fully hydrated film mass (W_h_). The water absorption capacity was subsequently calculated using the following equation.


(5) $$Absorption\;capacity\;(\%)=\frac{W_{h}-W_{s}}{W_{s}}\times 100\%$$

#### 4.3.8. Water Vapour Permeability (WVPt)

The determination was carried out using a gravimetric method following the procedure reported by [32], with some modifications. Environmental conditions were established to generate a humidity gradient ranging from 52.8% RH to 100% at 25 °C. Films exhibiting no physical defects were selected for water vapour permeability (WVPt) testing. Payne permeability cups filled with distilled water were employed, thereby exposing one side of each film to 100% RH. These assemblies were subsequently placed within humidity-controlled cabinets maintained at 25 °C, with the internal relative humidity (52.8%) regulated by means of supersaturated magnesium nitrate solutions. Furthermore, to accommodate the practical application of the films in products characterised by high water activity, the free surface of each film during its formation was subjected to a lower relative humidity. The cups containing the films were weighed at regular intervals using a precision analytical balance with a sensitivity of 0.0001 g. Once a steady-state measurement condition had been attained, the water vapour transmission rate (WVPt) was derived from the slope of the regression curve plotting weight loss against time. This value was then normalised by dividing by the film area. All measurements were conducted in triplicate, and the results are presented as the mean value accompanied by the corresponding standard deviation.

#### 4.3.9. Water and Oil Contact Angle

A film sample was placed on a horizontal white background surface to ensure a uniform measurement area. A 5 μL droplet of distilled water or soybean oil was carefully deposited onto the film surface using a calibrated micropipette. For the water measurements, the distilled water was coloured with a trace amount of food-grade dye to improve visual contrast between the droplet and the film surface during image acquisition. The dye concentration was minimal and used only for visualisation purposes, without significantly affecting the physicochemical properties of the liquid. Images were captured 30 s after droplet deposition using a digital camera positioned at a fixed distance of 20 cm from the sample and aligned with the droplet profile. The contact angle was determined by analysing the droplet shape using Goniotrans software (version 1.4.0, developed by Felipe Gordillo, Elche, Spain), which calculates the angle formed between the tangent at the liquid–solid interface and the baseline of the droplet according to the sessile drop method. Measurements were performed in triplicate for each formulation, and results were expressed as mean ± standard deviation [33].

#### 4.3.10. Thermal and Crystalline Properties: Study by Thermogravimetric Analysis (TGA), Differential Scanning Calorimetry (DSC), and X-Ray Diffraction (XRD)

For the analysis of thermal and crystalline properties, the methodology developed by [34] was employed. TGA curves were obtained using a thermogravimetric analyser (TA^®^ Instruments, model Q500, Hillhurst, Germany). Film samples, weighing between 7.8 and 24.1 mg, were heated from 25 °C to 700 °C at a rate of 10 °C/min under an air atmosphere (flow: 30 mL/min). Each film system was tested in triplicate. DSC thermograms were acquired using a DSC (TA^®^ Instruments, model Q2000, Hüllhorst, Germany). This analysis was conducted to determine the crystallisation and melting temperatures of the films, as well as their crystallisation and melting enthalpies. These parameters are related to the crystalline phase of the films. Additionally, the glass transition temperature and the change in heat capacity associated with the amorphous portion of the films were determined. X-ray diffraction (XRD) analysis was also conducted. This test was performed using an X-ray diffractometer (D8 Advance ECO, Bruker, Billerica, MA, USA). The samples were analysed over a 2θ range of 5–60°, using Cu Kα radiation (1.5418 Å) operated at 40 kV and 25 mA. The diffraction patterns were normalised to their maximum intensity to allow qualitative comparison among samples. FTIR. Functional groups were identified using spectroscopy (IR Prestige 21 Shimadzu, Kyoto, Japan). Spectra were recorded over a wavenumber range of 4500–500 cm^−1^, averaging 20 scans at a resolution of 4 cm^−1^.

#### 4.3.11. Microstructure

Cross-sectional images of the films were obtained by means of scanning electron microscopy (SEM) using a TOPCON SM-510 instrument (Topcon Corporation, Tokyo, Japan). The specimens were sputter-coated with gold and affixed to double-sided carbon tape. An accelerating voltage of 15 kV was employed, and observations were carried out at magnifications of 1000×, 1500×, and 2000×.

#### 4.3.12. Statistical Analysis

For the statistical treatment of the data, a one-way analysis of variance (ANOVA) was performed. In cases where statistically significant differences were detected (*p* < 0.05), multiple comparisons among sample means were carried out using Fisher’s least significant difference (LSD) post hoc test. All statistical calculations were performed using Statgraphics Centurion 19 (version 19.7.01; Manugistics Corp., Rockville, MD, USA).

## Figures and Tables

**Table 1:** Mean values and standard deviations of gloss parameters (GU) and colour parameters (lightness (L), red/green (a), yellow/blue (b*), chroma (C), and hue angle (h, °)) of the studied film.

Formulations	Gloss at 60°	Colour Parameters					ΔE
		L*	a*	b*	C	h	
Gel-c	67.43 ± 0.3 ^a^	80.7 ± 0.2 ^ab^	4.4 ± 0.1 ^c^	32.2 ± 0.1 ^d^	32.5 ± 0.3 ^c^	82.8 ± 0.6 ^bc^	-
Gel-Cy1	45.29 ± 0.8 ^c^	75.3 ± 0.5 ^c^	7.7 ± 0.01 ^a^	44.4 ± 0.2 ^a^	44.1 ± 0.01 ^a^	80.3 ± 0.6 ^c^	12.8 ± 0.1 ^a^
Gel-Cy2	55.86 ± 0.8 ^b^	83.5 ± 0.6 ^a^	5.1 ± 0.1 ^b^	23.7 ± 0.1 ^e^	24.1 ± 0.02 ^d^	95.0 ± 0.7 ^a^	8.3 ± 0.1 ^b^
Gel-Gt1	28.43 ± 0.4 ^d^	84.7 ± 0.8 ^a^	4.5 ± 0.2 ^c^	40.5 ± 0.1 ^b^	40.6 ± 0.2 ^b^	90.1 ± 0.2 ^ab^	8.6 ± 0.8 ^b^
Gel-Gt2	30.71 ± 0.4 ^d^	75.7 ± 0.1 ^bc^	4.4 ± 0.3 ^c^	34.4 ± 0.2 ^c^	13.2 ± 0.6 ^e^	91.8 ± 0.3 ^a^	5.3 ± 0.9 ^c^

Different superscript letters indicate statistically significant differences (*p* < 0.05) among the formulations.

**Table 2:** Mean values and standard deviations of thickness (μm), water vapour permeability (WVPt, g·mm/kPa·h·m^2^), swelling degree (Hwg, g water/g dry film), moisture content (Xwg, g water/g dry film), and water absorption capacity (Wcag, g dry film/g wet film) of the studied films.

Formulations	Thickness	WVPt	Hwg	Wcag	Xwg
Gel-c	215.7 ± 0.06 ^c^	0.05 ± 0.01 ^a^	3.96 ± 0.02 ^b^	0.46 ± 0.06 ^a^	0.108 ± 0.45 ^a^
Gel-Cy1	274.7 ± 0.03 ^b^	0.05 ± 0.01 ^a^	5.24 ± 0.08 ^a^	0.27 ± 0.07 ^ab^	0.107 ± 0.48 ^a^
Gel-Cy2	351.8 ± 0.06 ^a^	0.06 ± 0.01 ^a^	5.64 ± 0.05 ^a^	0.24 ± 0.02 ^b^	0.086 ± 0.08 ^b^
Gel-Gt1	219.6 ± 0.04 ^c^	0.05 ± 0.02 ^a^	3.60 ± 0.07 ^b^	0.59 ± 0.05 ^c^	0.072 ± 0.36 ^b^
Gel-Gt2	285.3 ± 0.04 ^b^	0.02 ± 0.01 ^b^	3.42 ± 0.01 ^b^	0.52 ± 0.02 ^c^	0.118 ± 0.11 ^a^

Different superscript letters indicate statistically significant differences (*p* < 0.05) among the formulations.

**Table 3:** Mean values and standard deviations of the water contact angle (CAw, °) and oil contact angle (CAo, °) of the studied monolayer and bilayer films (both surfaces).

Formulations	CAw	Cao
Gel-c	52.0 ± 1.4 ^d^	27.7 ± 1.5 ^b^
Gel-Cy1	62.7 ± 1.5 ^b^	31.7 ± 1.3 ^a^
Gel-Cy2	66.7 ± 1.1 ^a^	23.0 ± 1.0 ^c^
Gel-Gt1	59.0 ± 1.0 ^c^	31.6 ± 1.5 ^a^
Gel-Gt2	63.3 ± 1.5 ^b^	33.0 ± 1.0 ^a^

Different superscript letters indicate statistical differences (*p* < 0.05).

**Table 4:** Thermal properties of the formulated films: water release temperature (Td_1_, °C), initial degradation peak temperature (Td_2_, °C), and maximum degradation peak temperature (Td_3_, °C).

Formulations	Td1	Td2	Td3
Gel-c	118.15 ± 0.1 ^d^	245.9 ± 0.1 ^b^	303.60 ± 0.1 ^c^
Gel-Cy1	116.48 ± 0.3 ^d^	241.35 ± 0.2 ^c^	309.46 ± 0.4 ^b^
Gel-Cy2	129.48 ± 0.5 ^c^	245.35 ± 0.4 ^b^	314.76 ± 0.1 ^a^
Gel-Gt1	137.96 ± 0.1 ^b^	241.49 ± 0.3 ^c^	309.00 ± 0.1 ^b^
Gel-Gt2	159.9 ± 0.2 ^a^	247.07 ± 0.1 ^a^	309.04 ± 0.3 ^b^

Different superscript letters indicate statistically significant differences.

**Table 5:** Main diffraction peaks (P, °2θ) of the X-ray patterns of the studied hydrogels.

Formulation	P_1_ (~11°)	P_2_ (~20°)	P_3_ (~30°)	P_4_ (~31°)	P_5_ (~37°)	P_6_ (~40°)	P_7_ (~43)	P_8_ (~48°)	P_9_ (49°)	P_10_ (~58°)
Gel-c	11.0° ± 0.1 ^b^	21.0° ± 0.1 ^a^	29.4° ± 0.9 ^a^	31.1 ± 1.6	37.0° ± 1.2 ^a^	40.1 ± 1.6 ^a^	43.0° ± 1.2 ^b^	48.0° ± 1.4 ^a^	49.0° ± 1.3 ^a^	58.0° ± 1.4 ^b^
Gel-Cy1	12.0° ± 0.3 ^a^	20.0° ± 0.4 ^b^	28.5° ± 0.7 ^b^	—	36.0° ± 1.1 ^b^	39.0° ± 1.3 ^b^	43.2° ± 1.0 ^b^	47.5° ± 0.6 ^b^	48.4° ± 1.1 ^b^	57.3° ± 0.8 ^b^
Gel-Cy2	11.0° ± 0.5 ^b^	20.0° ± 0.1 ^b^	29.4° ± 1.2 ^a^	—	36.5° ± 1.4 ^b^	39.3° ± 1.6 ^b^	43.1° ± 1.6 ^b^	47.5° ± 1.8 ^b^	48.5° ± 1.6 ^b^	57.0° ± 0.4 ^b^
Gel-Gt1	11.0° ± 0.3 ^b^	20.5° ± 0.5 ^b^	29.5° ± 1.5 ^a^	—	36.1° ± 0.9 ^b^	39.5° ± 1.1 ^b^	43.2° ± 1.7 ^b^	47.8° ± 0.8 ^b^	48.7° ± 0.3 ^b^	57.5° ± 1.4 ^b^
Gel-Gt2	11.0° ± 0.6 ^b^	20.8° ± 0.3 ^b^	29.3° ± 1.3 ^a^	—	35.7° ± 1.5 ^c^	40.3° ± 1.3 ^a^	45.1° ± 0.7 ^a^	47.4° ± 0.3 ^b^	48.5° ± 1.2 ^b^	57.3° ± 1.1 ^b^

Different superscript letters indicate significant differences (*p* < 0.05) between the formulations.

**Table 6:** Band positions (cm^−1^) of the obtained hydrogels according to Fourier transform infrared (FTIR) spectra.

Wavenumber (cm^−1^)	Vibration°	Functional Group
3100–3500	O-H	Hydroxyl groups
2900–3000	C-H	Methyl groups
1600–1800	C=0	Carbonyl/Amide I
1560–1520	N-H y C-N	Amide II
1230–1270	C-N y C-O	Amide III
1150–1000	C-O-C	Glycosidic bonds

**Table 7:** Mass fractions of the formulations of the studied hydrogel films.

Formulations	Gelatine	Cysteine	Glutamine	Glycerol	Glutaraldehyde
Gel-c	0.86731	0.00000	0.00000	0.13010	0.00260
Gel-Cy1	0.85985	0.00860	0.00000	0.12898	0.00257
Gel-Cy2	0.85252	0.01705	0.00000	0.12788	0.00255
Gel-Gt1	0.85985	0.00000	0.00860	0.12898	0.00257
Gel-Gt2	0.85252	0.00000	0.01705	0.12788	0.00255

## Data Availability

The data presented in this study are available on request from the corresponding author.
